# Treatment with selectin blocking antibodies after lengthening contractions of mouse muscle blunts neutrophil accumulation but does not reduce damage

**DOI:** 10.14814/phy2.12667

**Published:** 2016-01-05

**Authors:** Darcée D. Sloboda, Susan V. Brooks

**Affiliations:** ^1^ Department of Biomedical EngineeringUniversity of MichiganAnn ArborMichigan; ^2^ Department of Molecular and Integrative PhysiologyUniversity of MichiganAnn ArborMichigan

**Keywords:** Injury, lengthening contraction, muscle, neutrophil, selectin

## Abstract

P‐ and E‐selectins are expressed on the surface of endothelial cells and may contribute to neutrophil recruitment following injurious lengthening contractions of skeletal muscle. Blunting neutrophil, but not macrophage, accumulation after lengthening contractions may provide a therapeutic benefit as neutrophils exacerbate damage to muscle fibers, while macrophages promote repair. In this study, we tested the hypothesis that P‐ and E‐selectins contribute to neutrophil, but not macrophage, accumulation in muscles after contraction‐induced injury, and that reducing neutrophil accumulation by blocking the selectins would be sufficient to reduce damage to muscle fibers. To test our hypothesis, we treated mice with antibodies to block P‐ and E‐selectin function and assessed leukocyte accumulation and damage in muscles 2 days after lengthening contractions. Treatment with P/E‐selectin blocking antibodies reduced neutrophil content by about half in muscles subjected to lengthening contractions. In spite of the reduction in neutrophil accumulation, we did not detect a decrease in damage 2 days after lengthening contractions. We conclude that P‐ and/or E‐selectin contribute to the neutrophil accumulation associated with contraction‐induced muscle damage and that only a portion of the neutrophils that typically accumulate following injurious lengthening contractions is sufficient to induce muscle fiber damage and force deficits. Thus, therapeutic interventions based on blocking the selectins or other adhesion proteins will have to reduce neutrophil numbers by more than 50% in order to provide a benefit.

## Introduction

Skeletal muscle injuries are common and have a variety of causes, including physical trauma, extreme temperatures, toxin exposure, invasive surgery, ischemia/reperfusion and unloading/reloading. Muscles can also be injured by their own contractions (i.e., contraction‐induced injury), especially during lengthening contractions when muscles are stretched while activated. Regardless of the cause, the initial insult is followed by swelling and inflammation, degeneration and necrosis of damaged muscle fibers, and regeneration and repair. In the young and healthy, regeneration is typically rapid and complete, but advanced age, muscle disease, and particularly severe injury are associated with delayed or incomplete repair. Therefore, reducing the degree of damage after an injurious event is a worthwhile goal.

Accompanying the degeneration and necrosis of fibers in injured muscle is the accumulation of inflammatory cells in the tissue. Neutrophils increase in the muscle within hours to days of the initial injury (Tidball and Villalta 2010). Neutrophils can lyse muscle cells in vitro and damage membranes in vivo by mechanisms involving reactive oxygen species (Nguyen and Tidball 2003b; Nguyen et al. 2005), and preventing neutrophil infiltration after injury reduces force deficits and histological damage to muscle fibers (Walden et al. 1990; Brickson et al. 2003; Pizza et al. 2005; Lockhart and Brooks 2008), suggesting that neutrophils contribute to muscle fiber damage. Subsequent to the rise in neutrophils, pro‐inflammatory macrophages begin to accumulate in injured muscle. Pro‐inflammatory macrophages are also capable of lysing muscle cells in vitro (Nguyen and Tidball 2003a). However, in vivo studies suggest that these cells are ultimately beneficial to the repair process, as evidenced by observations that reducing the number of invading macrophages delays the clearance of necrotic fibers, reduces the number and size of regenerating fibers, and increases fibrosis and fat relative to controls (Summan et al. 2006; Arnold et al. 2007; Segawa et al. 2008; Wang et al. 2014). Thus, preventing or blunting the neutrophil response while keeping the macrophage response intact may protect the muscle from unnecessary damage without interfering with repair, and provide a therapeutic benefit in cases of delayed or incomplete repair.

Methods explored to reduce neutrophil accumulation consist of systemic approaches such as depleting cells from the circulation (Lowe et al. 1995; Lockhart and Brooks 2008) or genetically knocking out genes for proteins involved in neutrophil migration out of blood vessels (Frenette et al. 2003; Pizza et al. 2005). These are appropriate experimental approaches for proof‐of‐concept studies, but are not feasible as therapeutic interventions. One option for targeted therapeutic intervention is the administration of a compound to block the endothelial selectins, P‐ and E‐selectin. P‐ and E‐selectins are adhesion proteins expressed by activated vascular endothelial cells. Once expressed on the luminal surface of blood vessels, the selectins interact with corresponding ligands on neutrophils, “capturing” them from the rapidly flowing blood stream and enabling slow rolling along the vessel wall. Capture and slow rolling of neutrophils are initial steps in a well‐characterized cascade that culminates with neutrophil migration out of the blood vessel and into the surrounding tissue (Ebnet and Vestweber 1999). Genetic knockout of both P‐ and E‐selectin prevents neutrophil infiltration after an unloading/reloading injury yet keeps macrophage accumulation intact (Frenette et al. 2003), but the degree to which endothelial selectins contribute to neutrophil and macrophage accumulation after other types of muscle injury is unknown. In particular, whether reducing neutrophil accumulation after lengthening contractions by targeting the selectins is sufficient to reduce damage to muscle fibers has not been examined.

Therefore, in this study, we tested the hypothesis that endothelial selectins contribute to neutrophil, but not macrophage, accumulation in muscles after contraction‐induced injury, and that reducing neutrophil accumulation by blocking the selectins would be sufficient to reduce damage to muscle fibers. To test our hypothesis, we subjected extensor digitorum longus (EDL) muscles of mice to lengthening contractions in situ and treated the animals with antibodies to block binding of neutrophils to P‐selectin and E‐selectin. Two days after lengthening contractions, damage was assessed by functional measures and by histology, and neutrophil and macrophage accumulation was assessed by immunohistochemistry.

## Methods

### Animals

Twenty‐two male C57BL/6N mice, 3–5 months of age were purchased from Charles River Laboratories (Wilmington, MA) and housed in a specific pathogen‐free facility at the University of Michigan until experimentation. Between experimental procedures, mice were housed in a separate specific pathogen‐free return room. All animal use procedures were approved by the University of Michigan Committee on the Use and Care of Animals.

### In situ evaluation of contractile properties

Procedures for in situ evaluation of muscle contractile properties were based on previous studies (Brooks and Faulkner 1990; Koh and Brooks 2001). Each mouse was anesthetized with 3% isoflurane in oxygen delivered at a rate of 1 L/min. Anesthesia was maintained throughout in situ procedures with 2% isoflurane in oxygen and depth of anesthesia was confirmed by failure of the mouse to respond to tactile stimuli. Ophthalmic ointment was placed on the mouse's eyes to prevent corneal drying and trauma, and this was readministered throughout in situ procedures. The mouse was placed on a platform warmed to 37°C with a circulating water bath. The hind limb fur was removed with animal clippers followed by a minimal amount of fur removal cream. The skin was disinfected with chlorhexidine and a small incision was made at the ankle to expose the distal tendon of the EDL muscle. The small area of exposed tendon was kept moist throughout in situ procedures by frequent administration of sterile saline. Another small incision was made distal to the knee to expose the peroneal nerve. A secure knot was tied around the tendon with 6.0 braided silk suture. The hind limb was immobilized by pinching the knee and the foot with small clamps secured to the platform. Using the tails of the silk suture, the intact tendon was tied to the lever arm of a servomotor (300C‐LR‐FP; Aurora Scientific, Aurora, ON), which controlled the length of the muscle and measured the force generated.

A computer with custom‐designed software‐controlled stimulus pulses, the servomotor, and collected and stored force data. The EDL muscle was activated using a stimulator (701C; Aurora Scientific) and platinum electrodes placed under the peroneal nerve. A stimulus pulse duration of 0.2 msec was used for all contractions. Stimulation current and muscle length were adjusted in order to elicit maximum twitch force. Tetanic contractions of 200 msec duration were elicited with trains of pulses and the frequency of the pulses was increased until the force plateaued at the maximum isometric force (P_o_), typically at a frequency of 200 Hz. Finally, small adjustments in the ankle position were made to elicit maximum isometric tetanic force. The tetanic contractions were spaced 1 min apart to prevent fatigue. Optimal muscle length (L_o_), defined as the muscle length at which maximum isometric force is achieved, was measured with calipers using the knee to estimate the location of the proximal end of the EDL muscle. Optimal muscle fiber length (L_f_) was determined by multiplying L_o_ by the previously determined L_f_‐to‐L_o_ ratio of 0.45 (Brooks and Faulkner 1988).

### In situ lengthening contraction protocol

Following evaluation of contractile properties, the EDL muscle was exposed to a protocol of 75 lengthening contractions spaced 4 sec apart for a total duration of 5 min. Each contraction was 300 msec in duration. 100 msec after the onset of stimulation, near‐maximum isometric force was generated and a stretch of 20% strain relative to L_f_ was initiated. Muscles were lengthened at the appropriate rate (1 L_f_/s) to cause the peak of the stretch to coincide with the end of the tetanic stimulation. Ten minutes after the lengthening contraction protocol, the muscle was relengthened to achieve maximum twitch tension and P_o_ was remeasured. The small incisions at the ankle and distal to the knee were closed with 7.0 sterile monofilament nylon suture and bathed with povidone‐iodine solution, and mice were monitored until they recovered from anesthesia.

### Administration of blocking antibodies

Approximately 1 h following in situ lengthening contractions, antibodies were administered via intraperitoneal injection(s). Antibodies were administered *after* lengthening contractions, since the intervention was designed to interfere with the inflammatory response that occurs subsequent to the initial injurious event. The specific time point of 1 h following the lengthening contractions was chosen to precede the bulk of neutrophil migration into injured muscle (Tidball and Villalta 2010) and allow for the completion of surgical procedures. Mice received either tandem injections of rat anti‐mouse monoclonal antibodies specific for P‐selectin (200 *μ*g, clone RB40.34; BD Pharmingen, San Diego, CA, 553741) and E‐selectin (200 *μ*g, clone 9A9, generously provided by Dr. Klaus Ley; La Jolla Institute for Allergy & Immunology) or a single injection of irrelevant isotype control antibodies (400 *μ*g, A110‐1; BD Pharmingen, 559157). Uninjected mice served as an additional control group.

The blocking function of RB40.34 and 9A9 has been demonstrated in many studies in vitro and in vivo. In vitro, both antibodies prevent attachment of myeloid cells to their respective selectins (Bosse and Vestweber 1994; Ramos et al. 1997). In vivo, RB40.34 alone or together with 9A9 prevents cytokine‐induced leukocyte rolling along blood vessel walls, and both antibodies reduce chemically induced neutrophil migration into the peritoneal cavity (Bosse and Vestweber 1994; Kunkel et al. 1997; Ramos et al. 1997; Thorlacius et al. 1997; Kanwar et al. 1998; Eriksson 2008). RB40.34 was detected on platelets in the blood 3 h after a single intraperitoneal injection, and platelets with bound RB40.34 were detected up to 7 days after injection when a dose of 200 *μ*g was administered (Phillips et al. 2003). Therefore, this dose of RB40.34 and 9A9 was used in this study to provide blocking coverage over the time period studied.

### In vitro evaluation of contractile properties

Two days following administration of the lengthening contraction protocols, mice were again evaluated for P_o_. This time point was chosen because preliminary experiments indicated that neutrophil content peaked in injured muscles 2 days after the contraction protocol used in this study and subsequently rapidly declined. Procedures for the in vitro evaluation of EDL contractile properties have been previously published (Brooks and Faulkner 1988). Each mouse was anesthetized with an intraperitoneal injection of Avertin (tribromoethanol, 250 mg/kg) (chemical components from Sigma‐Aldrich, St. Louis, MO). After the mouse was unresponsive to a tactile stimulus, the injured EDL muscle was isolated from the hind limb of the mouse. 5‐0 silk suture was tied to the proximal and distal tendons of the muscle, and the muscle was placed into a chamber containing Krebs Mammalian Ringer solution composed of (in mmol/L): 137 NaCl, 5 KCl, 2 CaCl_2_·2H_2_O, 1 MgSO_4_·7H_2_O, 1 NaH_2_PO_4_, 24 NaHCO_3_, 11 glucose, 0.03 tubocurarine chloride (chemicals from Sigma‐Aldrich). The solution was maintained at 25°C and bubbled with 95% O_2_–5% CO_2_ to maintain a pH of 7.4. The proximal tendon was attached to a stationary object and the distal tendon was attached to a force transducer (BG‐50; Kulite Semiconductor Products, Leonia, NJ). Muscle activation was accomplished by electric field stimulation via a high‐power current stimulator (701C; Aurora Scientific) and parallel plate electrodes.

A computer and custom‐designed software‐controlled stimulus pulses and collected and stored force data. Stimulus pulses of 0.2 msec in duration were used for all contractions. Stimulation current and the muscle length were adjusted in order to elicit maximum twitch force. A digital caliper was used to measure L_o_. Muscles were held at L_o_ and tetanic contractions of 300 msec in duration were elicited with trains of pulses. The frequency of the pulses was increased until the force plateaued at P_o_, typically at frequencies from 150 to 200 Hz. The tetanic contractions were spaced 1 min apart to prevent fatigue. Optimal muscle fiber length (L_f_) was determined as previously mentioned. Force deficit was defined as the difference between the P_o_ measured immediately prior to lengthening contractions and the P_o_ measured 2 days after lengthening contractions expressed as a percentage of the preinjury P_o_. Following evaluation of the injured EDL, the contralateral EDL was removed and evaluated using an identical procedure. Muscles were trimmed of their tendons and weighed. Muscles were then immersed in Tissue Freezing Medium (Electron Microscopy Sciences, Hatfield, PA) and frozen in isopentane cooled by liquid nitrogen. The mouse was killed with an overdose of Avertin followed by induction of a bilateral pneumothorax.

### Histology and immunohistochemistry

Extensor digitorum longus muscles were cut into 10 *μ*m thick sections on a cryostat. The sections were fixed in cold acetone and stained with Hematoxylin (Ricca Chemical Company, Arlington, TX) and Eosin Y (EMD Millipore, Billerica, MA). Stained sections were imaged with a Nikon E‐800 light microscope. The number of damaged fibers was counted per section and expressed as a percentage of the total number of fibers. Damaged fibers included those with a swollen appearance, pale or variable staining, and obvious infiltration of inflammatory cells (Koh and Brooks 2001).

Muscle sections were analyzed for neutrophil and macrophage content using immunohistochemistry. Sections were fixed in cold acetone and air dried. Sections were exposed to BLOXALL solution to block endogenous peroxidase activity followed by 10% normal rabbit serum in PBS (Phosphate Buffer Solution; Fisher Scientific, Pittsburgh, PA) to block nonspecific binding of subsequent antibodies. Neutrophils were detected with a rat anti‐mouse Gr‐1 antibody (clone RB6‐8C5, 1:50 dilution; BD Pharmingen, 550291) and macrophages were detected with a rat anti‐mouse CD68 antibody (clone FA‐11, 1:1000; AbD Serotec, Raleigh, NC, MCA1957) diluted in PBS containing 10% rabbit serum. After incubation in primary antibodies, sections were exposed to biotinylated mouse adsorbed anti‐rat IgG in PBS containing 10% rabbit serum. Following exposure to the secondary antibodies, the sections were treated with the VECTASTAIN Elite ABC reagent containing peroxidase followed by the peroxidase substrate 3, 3′‐diaminobenzidine (ImmPACT DAB). The sections were rinsed with PBS after each step except after the treatment with rabbit serum. All reagents were from Vector Laboratories (Burlingame, CA) unless stated otherwise. In the periphery, neutrophils express high levels of Gr‐1 while monocytes express low or no levels of Gr‐1 (Lagasse and Weissman 1996). Therefore, dark brown cells indicating high levels of Gr‐1 were counted as neutrophils while the occasional light brown cell of similar size was not counted. CD68 is expressed on macrophages and labels early‐invading macrophages that accumulate in injured skeletal muscle (Rabinowitz and Gordon 1991; Holness et al. 1993; Deng et al. 2012). An image analysis program (Image J) was used to count the number of neutrophils (Gr‐1+ cells) or macrophages (CD68+ cells) in one transverse section from the middle of each muscle. The same program was used to calculate the area of each section, and neutrophil or macrophage content was expressed per mm^3^ of muscle.

The location of neutrophils relative to blood vessels was also analyzed using immunohistochemistry. Neutrophils were labeled first according to the methods described in the preceding paragraph, except that the normal rabbit serum was replaced with Carbo‐Free Blocking Solution, which does not contain glycoproteins that can interfere with subsequent lectin‐based detection. To label blood vessels, muscle sections were first treated with solutions from an Avidin/Biotin Blocking Kit to block endogenous avidin and biotin. Sections were incubated in Carbo‐Free Blocking Solution to block nonspecific binding of the lectin. The sections were then treated with a biotinylated Griffonia (Bandeiraea) Simplicifolia Lectin I (GSL I, 10 *μ*g/mL in PBS). GSL I labels all blood vessels in skeletal muscle (Hansen‐Smith et al. 1988). The biotinylated lectin was detected by exposing sections to a VECTASTAIN Elite ABC reagent containing peroxidase followed by a peroxidase substrate (ImmPACT SG). The sections were rinsed with PBS containing 0.05% Tween 20 (Sigma‐Aldrich) following the lectin and ABC reagent steps. All reagents were from Vector Laboratories unless stated otherwise. The number of neutrophils detected within blood vessels was counted and expressed as a percentage of total neutrophils per section. This sequential labeling technique may not detect neutrophils within capillaries, rather within larger vessels only.

### Blood analysis

Blood was collected via a scalpel nick of the lateral tail vein (50–100 *μ*L per day). Whole blood was analyzed with a cell counter (Hemavet 950FS; Drew Scientific, Dallas, TX) to determine circulating white blood cell counts.

### Statistics

SigmaPlot (Systat Software, Inc., San Jose, CA) was used for statistical analysis of the data. Experimental groups were compared with a Student's *t*‐test unless otherwise indicated, with significance set a priori at *P* < 0.05. Data are expressed as means ± 1 SEM. Sample sizes (*n*) reflect the number of animals examined.

## Results

**Figure 1:**
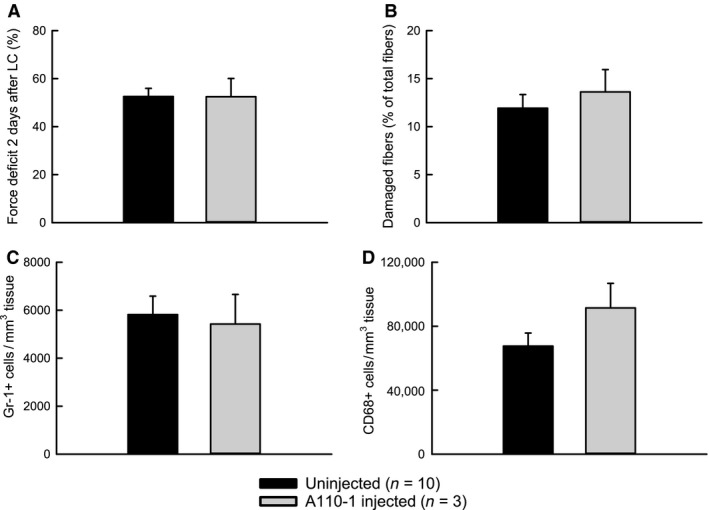
Injection of irrelevant control antibody A110‐1 has no significant effect on damage or inflammatory cells in muscles 2 days after lengthening contractions. Force deficit (A). Damaged fibers (B). Neutrophils (Gr‐1+ cells) (C). Macrophages (CD68+ cells) (D).

There were no differences among the experimental groups for body weight, maximum isometric force generation by the EDL muscle prior to lengthening contractions, or force deficit 10 min after the lengthening contractions (Table 1). Injecting the irrelevant control antibody A110‐1 had no significant effect on any of the variables measured (selected variables shown in Fig. 1). Therefore, the A110‐1 injected group was pooled with the uninjected group to make one “control” group.

**Table 1:** Characteristics of experimental groups prior to injections

Group	Sample size	Body weight (g)	Isometric force in situ (mN)	Immediate force deficit (%)
Uninjected	10	30.0 ± 0.6	414 ± 12	52 ± 3
A110‐1 injected	3	31.3 ± 1.4	411 ± 2	48 ± 6
RB40.34/9A9 injected	8	29.1 ± 0.5	395 ± 7	52 ± 4

Body weight, initial force generation of EDL muscles (measured prior to lengthening contractions), and immediate force deficit (measured 10 min after lengthening contractions) were not different among experimental groups as determined by one‐way analysis of variance tests. Values are means ± 1 SEM.

**Figure 2:**
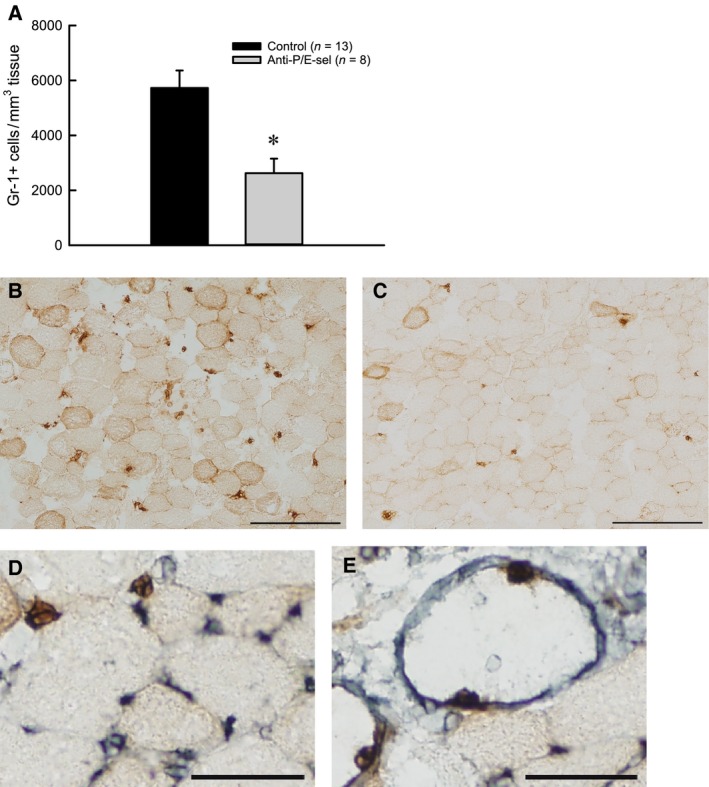
Treatment with blocking antibodies against P/E‐selectin decreases neutrophil content in muscles 2 days after lengthening contractions. Neutrophils (Gr‐1+ cells) *P = 0.005 by Mann–Whitney Rank Sum Test (A). Representative partial section of muscle from uninjected mouse (B) and mouse injected with blocking antibodies (C) with scale bars = 200 *μ*m. Examples of neutrophil (Gr‐1+ cells, brown) and blood vessel (GSL I, gray) co‐labeling with scale bars = 50 *μ*m (D, E). Neutrophils outside of vessels (D). Neutrophils on the inner surface of large vessels (E).

As hypothesized, treatment with P/E‐selectin blocking antibodies reduced neutrophil content by about half in muscles subjected to lengthening contractions (Fig. 2A). To assess whether the neutrophils that were present in the muscle in spite of blocking antibody treatment had indeed migrated out of the circulation, we examined neutrophil localization relative to blood vessels (Fig. 2D–E). Regardless of the experimental group, only about 5% of neutrophils were clearly inside blood vessels (control: 6 ± 1% vs. anti‐P/E‐sel: 5 ± 2%), suggesting that most of the neutrophils we detected were extravascular.

**Figure 3:**
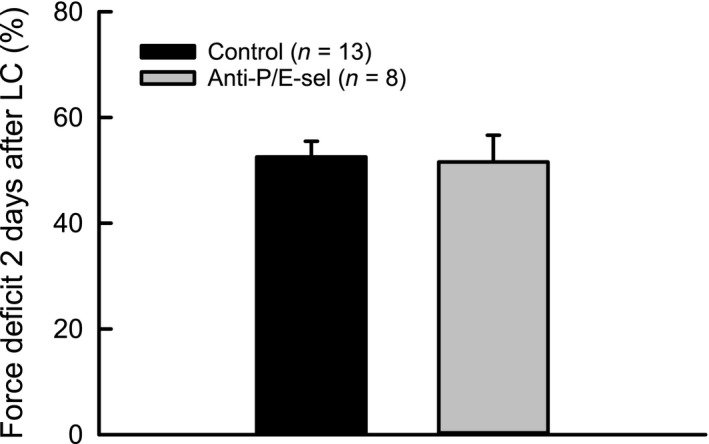
Treatment with blocking antibodies against P/E‐selectin does not significantly decrease muscle damage 2 days after lengthening contractions, as assessed by force deficit.

**Figure 4:**
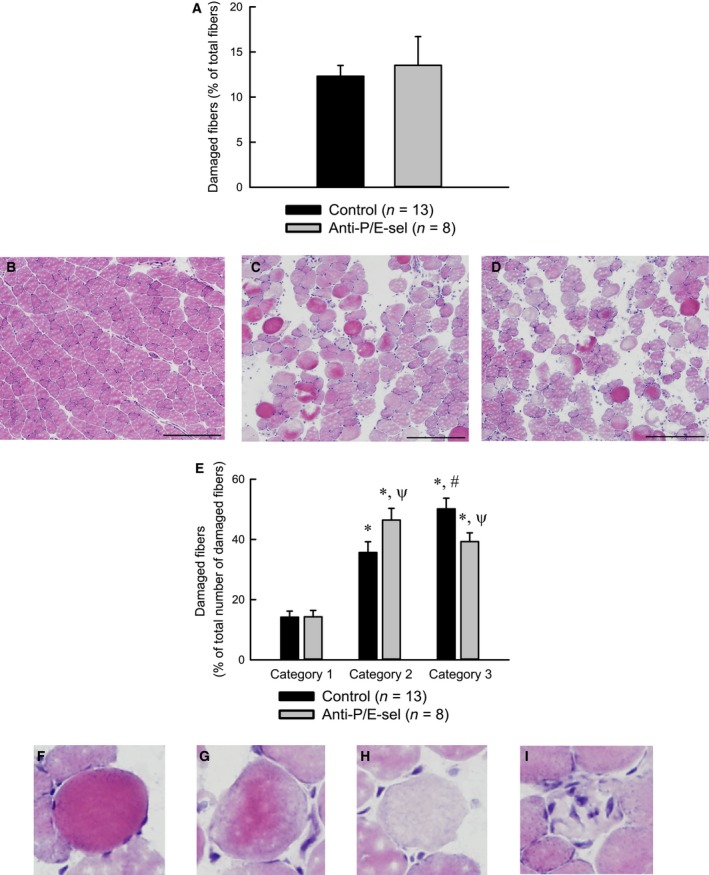
Treatment with blocking antibodies does not reduce the total number of damaged fibers, but reduces the percentage of damaged fibers that are invaded by inflammatory cells, 2 days after lengthening contractions. Total damaged fibers expressed as a percentage of the total number of fibers in a muscle section (A). Representative partial section of muscle from uninjured mouse (B), uninjected mouse (C), and mouse injected with P/E‐selectin blocking antibodies (D). Damaged fibers expressed as a percentage of the total number of damaged fibers in a muscle section (E). Damaged fibers fell into one of three categories. Category 1 consisted of round and often swollen fibers stained dark with eosin Y. Category 2 consisted of fibers with variable or pale staining with eosin Y. Category 3 consisted of fibers similar to those in category 2, but with several nuclei within the fiber, assumed to be inflammatory cells. *Significantly different from category 1 within experimental group. #Significantly different from category 2 within experimental group. *ψ*Significantly different from control within category. Significance was determined by a two‐way analysis of variance (*P* < 0.05) (E). Magnified views of fibers from C and D (F–I). Examples of category 1 (F), category 2 (G, H) and category 3 (I) fibers. Scale bars = 200 *μ*m.

**Figure 5:**
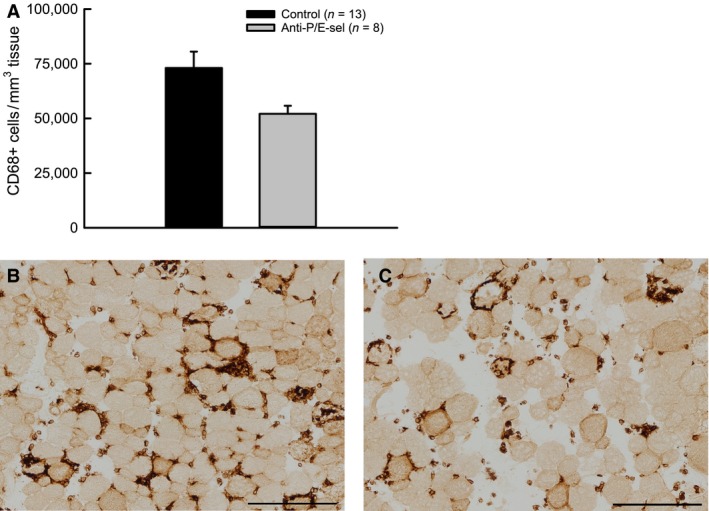
Treatment with blocking antibodies against P/E‐selectin decreases macrophage content in muscles 2 days after lengthening contractions, although the decrease is not statistically significant (*P* = 0.076 by Mann–Whitney Rank Sum Test). Macrophages (CD68+ cells) (A). Representative section of muscle from uninjected mouse (B) and mouse injected with blocking antibodies (C). Scale bars = 200 *μ*m.

Contrary to our hypothesis, the decrease in neutrophil content was not associated with a decrease in damage. The force deficit was approximately 50% for muscles of both treated and control mice (Fig. 3). Muscles of both groups also showed similar numbers of damaged fibers, with 10–15% of the fibers in a cross section showing morphological evidence of injury (Fig. 4A). Although the number of damaged fibers was not different among experimental groups, the characteristics of the damaged fibers were different (Fig. 4E). For muscles of mice injected with the blocking antibodies, fewer fibers showed signs of inflammatory cell infiltration and more fibers were present with variable or pale staining. Another finding contrary to our hypothesis was the observation that macrophage content showed a trend (*P* = 0.076) toward a reduction by approximately one‐third in muscles of treated compared with control mice (Fig. 5).

**Figure 6:**
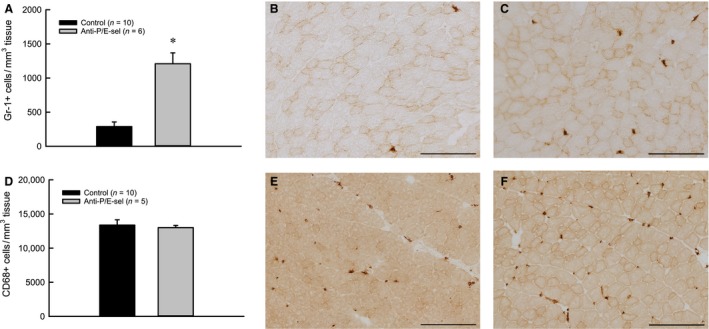
Treatment with blocking antibodies against P/E‐selectin increases neutrophil, but not macrophage content after 2 days, in contralateral muscles that have not been subjected to lengthening contractions. Neutrophils (Gr‐1+ cells) *P < 0.001 (A). Representative partial section of muscle from uninjured mouse (B) and mouse injected with P/E‐selectin blocking antibodies (C), Gr‐1 labeled. Macrophages (CD68+ cells) *P* = 0.854 by Mann–Whitney Rank Sum Test (D). Representative partial section of muscle from uninjured mouse (E) and mouse injected with P/E‐selectin blocking antibodies (F), CD68 labeled. Scale bars = 200 *μ*m.

**Figure 7:**
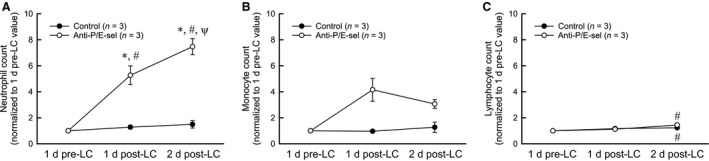
Circulating levels of major populations of white blood cells before and after lengthening contractions (LC). Note that injection of blocking antibodies occurred within an hour of lengthening contractions. Neutrophils increased significantly with blocking antibody treatment *significantly different from control, #significantly different from 1 day pre‐LC value *ψ*significantly different from 1 day post‐LC value (A). Monocytes increased with blocking antibody treatment but the increase did not reach significance (*P* = 0.066) (B). Lymphocytes increased 2 days after lengthening contractions in both experimental groups # significantly different from 1 day pre‐LC value (C). Data was analyzed by two‐way repeated measures analysis of variance tests (significance set at *P* < 0.05).

An unexpected finding was an effect of treatment with P/E‐selectin blocking antibodies on the contralateral muscles that were not subject to lengthening contractions (Fig. 6). Although force generation of contralateral control muscles was not affected by the treatment with the selectin blocking antibodies (control: 258 ± 5 mN/mm^2^ vs. anti‐P/E‐sel: 248 ± 6 mN/mm^2^) nor was there any morphological evidence of damaged fibers (control: 0.13 ± 0.07% vs. anti‐P/E‐sel: 0.23 ± 0.10%), neutrophil content was increased by more than fourfold in contralateral uninjured control muscles of mice treated with blocking antibodies (Fig. 6A). The majority of the 1210 ± 159 Gr‐1+ cells/mm^3^ found in contralateral control muscles appeared to be extravascular, as only 13 ± 5% of these cells were found within blood vessels. Although neutrophils were elevated in contralateral muscles of treated mice, the levels did not reach those observed in injured muscles of treated mice (contralateral: 1210 ± 159 Gr‐1+ cells/mm^3^ tissue vs. injured: 2624 ± 530 Gr‐1+ cells/mm^3^ tissue, *t*‐test, *P* = 0.045). Blood analysis revealed that the blocking antibody treatment dramatically increased circulating neutrophils (Fig. 7A). Treatment with P/E‐selectin blocking antibodies also increased circulating monocytes (Fig. 7B), although the difference was less dramatic than for neutrophils and did not reach significance (effect of blocking antibody treatment, *P* = 0.066). The trend toward elevated numbers of circulating monocytes was not associated with increased macrophage content in contralateral muscles (Fig. 6D).

## Discussion

The major implication of this study is that endothelial selectins (P‐ and/or E‐selectin) contribute to neutrophil accumulation after contraction‐induced injury. Support for this conclusion is provided by our observation that treating animals with blocking antibodies for P‐ and E‐selectin reduced the number of neutrophils in sections of injured muscles. Our results also indicate that reducing neutrophil accumulation by as much as 50% is not sufficient to reduce damage after contraction‐induced injury, as evidenced by the observations that the treatment and accompanying blunted accumulation of neutrophils did not result in a reduction of the force deficit or in the number of fibers showing histological evidence of damage. Our study was not decisive on the question of whether or not blocking the endothelial selectins left macrophage accumulation intact. Muscles of mice treated with blocking antibodies showed two‐thirds as many macrophages following lengthening contractions compared with the numbers observed in untreated mice, but the decrease did not reach statistical significance. Therefore, the remainder of the discussion will focus primarily on neutrophils.

The finding that endothelial selectins contribute to neutrophil accumulation after contraction‐induced injury was not known, but was expected. Many studies have demonstrated a role for the selectins in leukocyte capture and rolling along blood vessel walls in the cremaster muscle (Kunkel et al. 1997; Thorlacius et al. 1997; Kanwar et al. 1998; Jung and Ley 1999; Eriksson 2008). In addition, neutrophil accumulation in soleus muscle after unloading followed by reloading was reduced in mice deficient in P‐ and E‐selectin, suggesting that the endothelial sections contribute to neutrophil accumulation in this injury model (Frenette et al. 2003). Despite these previous investigations of selectin function in skeletal muscle, neutrophils can accumulate in tissues using adhesion molecules other than P‐ and E‐selectin, such as L‐selectin or vascular cell adhesion molecule 1 (Ley et al. 1995; Jung and Ley 1999; Lozano et al. 1999; Hickey et al. 2000; Eriksson 2008), and the involvement of endothelial selectins in neutrophil accumulation following contraction‐induced injury had not been demonstrated prior to this study.

Our finding that treatment with blocking antibodies reduced neutrophil accumulation in injured muscles, but did not reduce damage is unexpected but compatible with existing studies. Several studies examining damage after lengthening contractions found that effectively preventing neutrophil accumulation to a great extent (80–90% reduction in neutrophils) reduced force deficit and histological damage after injury caused by lengthening contractions, with specific reductions in damage ranging from approximately 30–80% (Brickson et al. 2003; Pizza et al. 2005; Lockhart and Brooks 2008). However, a reduction in damage by neutrophil depletion is not a universal finding. In one study, >90% of circulating neutrophils were depleted with antisera prior to lengthening contractions of hind limb muscles, yet no reduction in force deficit or in the quantity of damaged fibers relative to controls was observed (Lowe et al. 1995). Nevertheless, the majority of studies to date suggest that the damage after lengthening contractions is at least in part mediated by neutrophils. In light of these studies, the most defensible interpretation of our findings is that the residual neutrophil accumulation in the presence of blocking antibody treatment, about 50% of levels observed in untreated animals, was sufficient to induce neutrophil‐mediated damage in injured muscles.

While the blocking antibody treatment did not affect the total number of damaged fibers, the characteristics of damaged fibers were altered by the treatment (Fig. 4E). Increased intracellular calcium after muscle injuries can cause hypercontractions of portions of muscle fibers and leave adjacent portions of the same fibers barely visible or missing (Carpenter and Karpati 1989; Fridén and Lieber 1998). Thus, dark, swollen fibers (Category 1) or fibers with variable staining (Category 2) could represent portions of fibers that were hypercontracted, and pale fibers (Category 2) could represent segments of fibers that were adjacent to hypercontracted regions or fibers containing degraded contractile proteins, perhaps as a result of calcium‐sensitive proteases (Belcastro et al. 1998). Data from a parallel experiment using untreated mice showed that 5 days after lengthening contractions, the number of Category 1 and 2 fibers declined while the number of Category 3 fibers (pale fibers containing several nuclei, assumed to be inflammatory cells) increased (data not shown), suggesting that over the course of the degenerative phase after injury, fibers show signs of damage and degeneration and are subsequently invaded by inflammatory cells. Thus, an interpretation of Figure 4E is that the blocking antibody treatment did not affect the number of fibers showing signs of degeneration or necrosis, but decreased the number of degenerating or necrotic fibers that were subsequently invaded by inflammatory cells. This finding could be explained by decreased neutrophil accumulation with antibody treatment, or it could be explained by the reduced macrophage accumulation with antibody treatment, as macrophages are frequently found within degenerating fibers 2 days after injury.

Both neutrophils and macrophages are thought to participate in the removal of necrotic tissue after injury. Teixeira et al. (2003) depleted neutrophils prior to toxin‐induced muscle injury and observed delayed clearance of necrotic fibers accompanied by fewer regenerating fibers and fibrosis. However, the impaired regeneration could have been due to the decrease in macrophage accumulation that was also observed (Teixeira et al. 2003) as other studies that have depleted only macrophages prior to muscle injury have found similar impairments in regeneration (Summan et al. 2006; Arnold et al. 2007). We did not examine whether the reduced neutrophil accumulation observed in this study ultimately had an impact on the removal of necrotic tissue or on regeneration and repair, but this is an important consideration for future studies.

Although our study was not decisive on the question of whether or not blocking the endothelial selectins left macrophage accumulation completely intact, we can say that macrophage accumulation was not dramatically altered with P‐ and E‐selectin blocking antibody treatment. This conclusion is consistent with a previous report that treatment with RB40.34 did not reduce accumulation of CD11b+ cells after lengthening contractions in mouse soleus muscles (Baker et al. 2004). Although CD11b is present on neutrophils, monocytes, and macrophages, the authors report very few neutrophils in their study. Therefore, their finding suggests that monocyte/macrophage accumulation is not dependent upon P‐selectin, with the caveat that they may have not seen an effect due to the small concentration of RB40.34 injected (20 *μ*g) or the targeting of only one selectin. Another report found that macrophage accumulation after unloading and reloading of mouse soleus muscle was not affected in P/E‐selectin deficient mice (Frenette et al. 2003). Overall, the data from the previous studies as well as this study suggest that macrophages accumulate in injured skeletal muscle predominantly by P/E‐selectin independent mechanisms. The trend toward decreased macrophage accumulation observed in this study could be due to the decrease in neutrophil accumulation, as macrophage recruitment is one proposed role for neutrophils after muscle injury (Teixeira et al. 2003).

We were surprised by the observation that treatment with P‐ and E‐selectin blocking antibodies increased neutrophil content in the contralateral muscles that were not subject to lengthening contractions. Blood analysis showed that treatment with blocking antibodies after injury dramatically increased the level of circulating neutrophils. The latter finding is consistent with other reports also showing elevated levels of circulating neutrophils in selectin‐deficient mice or in mice treated with blocking antibodies for P‐ and E‐selectin (Labow et al. 1994; Ramos et al. 1997; Frenette et al. 2003), and may reflect the displacement of marginated neutrophils from blood vessel walls. Therefore, the dramatic increase in circulating neutrophils observed in this study may have driven more neutrophils into the contralateral muscles and presumably into the injured muscles, as well. We did not see more macrophages in the contralateral muscles, perhaps because there was only a trend for an increase in circulating levels of monocytes. Despite the substantial increase in neutrophil content in the contralateral muscles, we saw no evidence that the presence of the neutrophils in the tissue had any damaging effects on the muscle as assessed by the maintenance of force‐generating capability and also by the lack of histological evidence of injury. The uncoupling between neutrophil accumulation and muscle injury implies that merely the presence of neutrophils is not sufficient to induce damage. Additional factors such as neutrophil transcriptional activity, degranulation, or reactive oxygen species production, perhaps induced by the injured tissue environment, may be responsible for neutrophil‐mediated exacerbation of damage to muscle fibers.

In summary, this study provides evidence that endothelial selectins (P‐ and/or E‐selectin) contribute to neutrophil accumulation after contraction‐induced injury, while macrophage accumulation appears to occur by mostly endothelial selectin‐independent mechanisms. In spite of the reduction in neutrophil accumulation, we did not detect a decrease in damage 2 days after lengthening contractions, suggesting that the levels of neutrophils in the muscles (50% of levels in untreated animals) were sufficient to induce damage. Although we failed to demonstrate a benefit by blocking the selectins, this therapeutic approach should not be completely ruled out. Changes in the timing of antibody administration or in the quantity of antibody delivered may have led to a positive outcome. Regardless of the method used to decrease neutrophil numbers in injured muscle, this study in conjunction with other reports suggest that interventions targeting the accumulation of neutrophils would have to reduce neutrophil numbers to very low levels in order to be effective. Determining the effect of these approaches on later stages of the repair process is also an important area of investigation. Therapies targeting adhesion molecules or specific functions of neutrophils will also have to consider the effect on the muscle at later stages of the repair process.

## Conflict of Interest

The authors have no conflicts of interest to report.
